# The TRIMendous Role of TRIMs in Virus–Host Interactions

**DOI:** 10.3390/vaccines5030023

**Published:** 2017-08-22

**Authors:** Sarah van Tol, Adam Hage, Maria Isabel Giraldo, Preeti Bharaj, Ricardo Rajsbaum

**Affiliations:** 1 Department of Microbiology and Immunology, University of Texas Medical Branch, Galveston, TX 77555, USA; savantol@utmb.edu (S.v.T.); arhage@utmb.edu (A.H.); migirald@utmb.edu (M.I.G.); prbharaj@utmb.edu (P.B.); 2 Institute for Human Infections and Immunity, University of Texas Medical Branch, Galveston, TX 77555, USA

**Keywords:** innate immunity, virus infection, tripartite motif (TRIM), E3-ubiquitin ligase, ubiquitin, viral antagonism, type-I interferons, unanchored polyubiquitin

## Abstract

The innate antiviral response is integral in protecting the host against virus infection. Many proteins regulate these signaling pathways including ubiquitin enzymes. The ubiquitin-activating (E1), -conjugating (E2), and -ligating (E3) enzymes work together to link ubiquitin, a small protein, onto other ubiquitin molecules or target proteins to mediate various effector functions. The tripartite motif (TRIM) protein family is a group of E3 ligases implicated in the regulation of a variety of cellular functions including cell cycle progression, autophagy, and innate immunity. Many antiviral signaling pathways, including type-I interferon and NF-κB, are TRIM-regulated, thus influencing the course of infection. Additionally, several TRIMs directly restrict viral replication either through proteasome-mediated degradation of viral proteins or by interfering with different steps of the viral replication cycle. In addition, new studies suggest that TRIMs can exert their effector functions via the synthesis of unconventional polyubiquitin chains, including unanchored (non-covalently attached) polyubiquitin chains. TRIM-conferred viral inhibition has selected for viruses that encode direct and indirect TRIM antagonists. Furthermore, new evidence suggests that the same antagonists encoded by viruses may hijack TRIM proteins to directly promote virus replication. Here, we describe numerous virus–TRIM interactions and novel roles of TRIMs during virus infections.

## 1. Introduction

Eukaryotes are constantly exposed to a variety of pathogens, including viruses. As with other environmental signals, viral invasion triggers tightly regulated intracellular signaling cascades to optimally respond to infection. Mammals enact both an innate and an adaptive immune response to identify an infecting pathogen, to clear the foreign agent, and to protect against subsequent invasion. A primary mechanism for fine-tuning molecular pathways is utilization of post-translational modifications. Altering the functional proteome influences protein interactions, transcriptional programs, translation, secretion, and cytoskeletal arrangement. A variety of molecules, including phosphates, sugars, lipids, or proteins, can be attached or removed enzymatically to modulate protein function. Post-translational modifications thus enable rapid and reversible regulation.

### 1.1. Ubiquitin

Ubiquitin is a key protein in orchestrating immune responses. Ubiquitin is a 76-amino-acid protein that associates either covalently or non-covalently with proteins [1,2,3]. Interaction of ubiquitin with its targets may influence subcellular localization, signaling, trafficking, protein stability, and cell cycle progression [1,2,3]. The addition of ubiquitin to a protein target is catalyzed by three classes of ubiquitin enzymes. The ubiquitin activating enzyme (E1) binds the C-terminal glycine of the ubiquitin molecule to its active-site cysteine in an ATP-dependent manner to form a high-energy thioester bond [1,2]. Interaction of the E1 with ubiquitin exposes a site to enable the recruitment of the next enzyme [4]. Via a trans-thiolation reaction, the ubiquitin is transferred from the E1 to the E2 active-site cysteine [1,2]. Once the ubiquitin is bound, an E3 ubiquitin ligase can interact with an E2-ubiquitin conjugase and assist in the transfer of the ubiquitin from the E2 onto a target protein [1,2]. When covalently attaching ubiquitin to a protein, most often the ubiquitin is covalently linked at the lysine (K) ε-amino group [1,2].

Since ubiquitin has seven lysine residues itself, K6, 11, 27, 29, 33, 48, and 63, the ubiquitin enzymes can coordinate the formation of covalently linked polyubiquitin (poly-Ub) chains [1,2]. In addition, ubiquitin molecules can be linked in a head-to-tail orientation in which the donor ubiquitin’s C-terminal glycine is linked to the acceptor ubiquitin’s methionine-1 amino group [1,2]. The E2 primarily determines the ubiquitin chain topology [4,5] while the E3 is more important in identification and recruitment of the appropriate target [4]. Ubiquitin can be covalently linked to a protein as a single ubiquitin at one site (monoubiquitination), single ubiquitin molecules at multiple lysine residues of the same target protein (multi-monoubiquitination), or a covalently linked chain of ubiquitin to a single lysine (poly-Ub) [1]. Additionally, ubiquitination enzymes can synthesize unanchored poly-Ub chains, which are not covalently attached to any other protein, and act as a ligand for proteins containing a ubiquitin-binding domain (UBD) [1,4,6,7]. In addition to the complexity of chain length and topology, phosphorylation and acetylation of ubiquitin has also been identified [8,9]. The unique combination of poly-Ub chain topologies, covalent or non-covalent modifications, and chain length variation allows for precise signaling regulation.

The host encodes a variety of mechanisms to enact, regulate, and interpret this complex ‘ubiquitin code.’ Ubiquitin modification can directly influence the target molecule through induction of a conformational change. Such changes may recruit other molecules, expose a subcellular localization sequence, or alter protein stability [1,3]. Recognition of ubiquitin by UBD-containing proteins is analogous to a receptor binding its ligand. The UBD-containing proteins may recognize a specific poly-Ub chain topology (e.g., UBD specifically recognizing K48-linked poly-Ub chains) and/or chain length (e.g., a UBD that interacts with monoubiquitin) and then recruit specific molecules to create a signaling complex [1,3,10]. Although some exceptions exist, different poly-Ub chain topologies are associated with particular effector responses. The best-characterized examples of chain topologies include K48-linked poly-Ub chains, which stereotypically target proteins for proteasome-mediated degradation, or K63-linked poly-Ub chains, which generally coordinate cell signaling complexes and subcellular localization [1,3]. Despite the canonical roles of these chain topologies, K48-linked poly-Ub chains have been noted to be involved in promoting signaling complex assembly [11] and K63-linked poly-Ub chains can mark protein targets for degradation [12]. To regulate the ubiquitin response, the host also encodes deubiquitinating enzymes (DUB) or ubiquitin specific proteases (USP), which can disassemble poly-Ub chains or coordinate the removal of ubiquitinated enzymes [1,3]. Overall, the host encodes the enzymes necessary to generate, respond to, and eliminate ubiquitin modifications.

### 1.2. Tripartite Motif (TRIM) Proteins

Tripartite motif proteins (TRIMs) are an E3 ligase family critical in many cellular functions, including the regulation and coordination of innate immunity and antiviral responses [6,7,13,14,15,16]. TRIMs are known to coordinate the ubiquitination of target proteins to assemble signaling complexes, mediate proteolytic degradation, alter subcellular localization, or modulate the host’s transcript or protein composition (reviewed in: [2,6,7,17]). TRIM proteins are such named due to their conserved RBCC domain. The RBCC domain includes a really interesting new gene (RING) E3 ligase domain (R), one or two B-box domains (B), and a coiled-coil domain (CC) [2,18]. Like other RING motif-containing E3 ligases, the RING domain of TRIMs usually mediates the interaction with ubiquitin-bound E2 via the zinc finger motifs [2,4,17]. These motifs are comprised of key cysteine and histidine residues that coordinate binding to two zinc ions and facilitate protein–protein interactions [2,4,17]. The function of the B-box is less well characterized, however studies suggest that this domain is important in coordinating TRIM self-association and protein–protein interactions, as well as promoting formation of higher-order TRIM oligomerization (reviewed in [17]). Since the B-box domain also contains a zinc finger motif, it has been suspected to confer E3 ubiquitin ligase activity to some TRIMs [19,20], although it does not appear to be a general feature of the TRIM family [21]. Some TRIMs include only a B-box 2 domain, while others encode a B-box 1 followed by a B-box 2 [17,18].

The coiled-coil domain is a hyper-helical structure that allows for dimerization and self-association of TRIMs [17,18,21,22]. Crystal structures and biochemical analyses of the coiled-coil regions of TRIM5α, TRIM20, TRIM25 and TRIM69 indicate that the TRIM dimers are formed by coiled-coil antiparallel helical structures [17,22,23,24,25], and bioinformatics analysis suggest that this could be a general characteristic of the TRIM family [22]. Often, dimerization of TRIMs via the coiled-coil domain and formation of higher order oligomerization through the B-box domain is critical for TRIM function and E3 ligase activity [17,21,23,26,27,28,29]. Furthermore, in some cases, the antiparallel nature of the coiled-coil dimers and the formation of higher order oligomers allows for dimerization of RING domains, which are also important for enzymatic activity [17,23]. However, the presence of one or two B-box domains, or the presence of different C-terminal regions, may affect dimerization and/or higher order oligomerization of TRIMs. In fact, the oligomerization mode of TRIM32 and TRIM25, which contain one and two B-box domains respectively, differs greatly and is critical for their function [21]. Prediction of TRIM structure and function is further complicated by differences in TRIM domain composition. For example, although TRIM members are characterized by the presence of an RBCC, some TRIM-like members, including TRIM14 and TRIM16, lack a RING domain within the RBCC [19,30].

The variable C-terminal region of TRIMs is responsible primarily for interaction with target proteins and subcellular localization [2,18,31]. To date, 11 classes of TRIM C-terminal domains have been characterized [7,17,18]. The most prevalent TRIM C-terminal domain is the B30.2, or PRY-SPRY, domain with approximately 40 members identified in humans [6,17,32]. TRIMs post-translational modifications [11,33,34,35,36,37], alternatively spliced isoforms [16,38,39], and heterodimerization [18,19] expand the diversity and functionality of this protein family. The expanding interest in TRIMs relates to the realization that these proteins play crucial roles in regulating responses to pathogens, autoimmune pathologies, and cancer.

The evolutionary history of TRIMs in vertebrates correlates with the role of this E3 ligase family in regulating responses to viral infection. Though present in invertebrates, the TRIM family is greatly diversified in vertebrates coinciding with the evolution of the interferon (IFN) pathway and adaptive immune system [32,38]. Further, several TRIMs are clustered within chromosomes [38,39,40]. The clustering of closely related TRIMs suggests they evolved due to gene duplication [38,39,40,41]. The close proximity of several TRIMs, particularly those with a B30.2 domain, near known immune genes such as HLA (Human Leukocyte Antigen) [38,39,40] supports immune regulation as a TRIM-mediated function. Additionally, the regulatory regions of several TRIMs include target sequences recognized by transcription factors involved in coordinating immune responses [42]. The species-specific expansion of B30.2-containing TRIMs [32,43] suggests that this group is specialized. In humans, the 10 TRIMs without murine orthologs all contain a B30.2 domain [32]. Additionally, the region encoding the C-terminus of many TRIMs is under positive selection [32,39,41,43,44]. The non-coding, transcriptional regulatory regions of more recently evolved TRIM genes are also under positive selection [42]. This species-specific pattern of positive selection of closely related TRIMs suggests that individual TRIMs play specific antiviral roles.

In addition to differential mRNA expression upon viral infection, several TRIM family members are intimately involved in the antiviral response. Type-I IFNs and other cytokines, such as pro-inflammatory cytokines induced via the NF-κB pathway, have been noted to differentially regulate the expression of a significant population of TRIMs [7,45,46,47,48,49,50,51]. Likewise, TRIM overexpression influences the transcription of type-I IFN, pro-inflammatory cytokines, and IFN-stimulated genes (ISGs) [7,16]. The roles of TRIMs in viral infection include intrinsic restriction of viral pathogens, positive regulation of immune pathways that promote viral clearance, and negative regulation of antiviral pathways to limit immunopathology [6,7,52]. The incorporation of TRIM antagonists into viral genomes exemplifies the importance of TRIMs in antiviral responses [53,54,55,56,57,58]. Here, we will focus on the role of TRIMs in the direct and indirect inhibition of viruses and novel mechanisms of viral-mediated antagonism and hijacking of TRIMs. Excellent reviews on the roles of TRIMs in autophagy, cancer, and other diseases have been recently published [59,60,61].

## 2. TRIM-Mediate Regulation of Antiviral Signaling

### 2.1. Introduction to Innate Antiviral Responses

**Figure 1:**
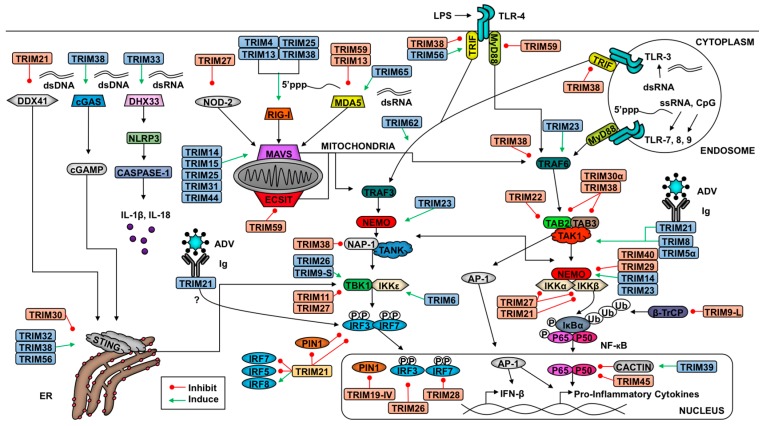
Regulation of pattern recognition receptor (PRR) signaling by tripartite motifs (TRIMs). TRIMs play an integral role in the positive and negative regulation of antiviral pathways. TRIMs can act as pathogen PRRs, as is the case for TRIM21 in the recognition of non-enveloped viruses bound by immunoglobulin (Ig). Additionally, these TRIMs can regulate the activation of other PRRs that recognize viral pathogen-associated molecular patterns (PAMPs) in the cytosol (DDX41 (DEAD-box helicase 41), cyclic GMP-AMP synthase (cGAS), DEAH-box helicase 33 (DHX33), nucleotide-binding oligomerization domain-containing protein 2 (NOD2), retinoic acid-inducible gene I (RIG-I), and melanoma differentiation-associated protein (MDA5)) and at membrane surfaces (toll-like receptors, TLRs). Downstream of the initial pattern recognition, TRIMs also influence the recruitment and interaction of adaptor molecules (stimulator of IFN genes (STING), mitochondrial antiviral signaling protein (MAVS), TGF-β-activated kinase 1(TAK1)/MAP3K7-binding protein (TAB) 2, Myeloid differentiation primary response gene 88 (MyD88), TIR-domain-containing adapter-inducing interferon-β (TRIF), NF-κB essential modulator (NEMO), nucleosome assembly protein (NAP-1), and tumor necrosis factor (TNF) receptor-associated factors (TRAF) family member-associated NF-κB activator (TANK)) and enzymes (TRAF3, TRAF6, TAK1, inhibitor of NF-κB (IκB) kinase (IKK) α,β,ε, TANK binding kinase 1 (TBK1)) to signaling complexes in order to activate transcription factors. This includes IFN regulatory factor (IRF)3 and IRF7, important in type-I interferon (IFN) signaling, and NF-κB, important in expression of pro-inflammatory genes, which regulate the expression of antiviral effectors. Type-I IFN production is critical for an effective antiviral response.

**Figure 2:**
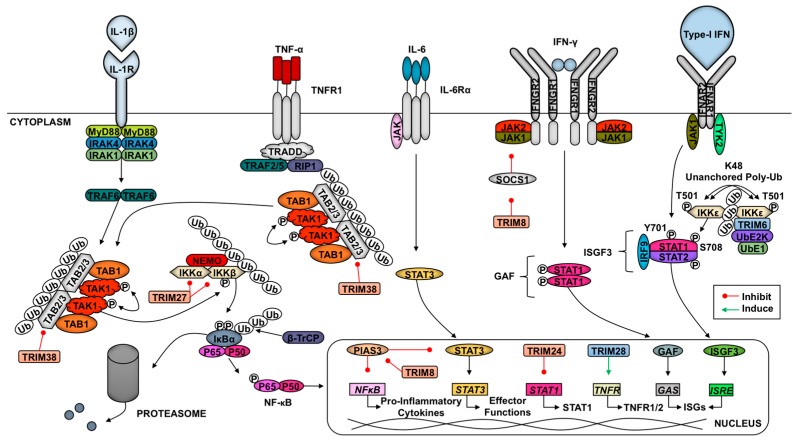
TRIMs in cytokine signaling. Downstream of the initial pathogen recognition and induction of pro-inflammatory cytokines, TRIMs can regulate their cytokine signaling pathways through interactions with cytokine receptor adaptors (TAB2/3) and enzymatic proteins (IKKα, IKKβ and IKKε) within the signaling complexes, the activity and stability of pathway negative regulators (Protein Inhibitor of Activated STAT 3 (PIAS3), suppressor of cytokine signaling (SOCS), and influence the transcription of various cytokine-effector genes (NF-κB-induced pro-inflammatory cytokines, signal transducer and activator of transcription (STAT)-induced genes, interferon stimulated genes (ISGs)) or cytokine signaling regulators (tumor necrosis factor (TNF) receptors (TNFR1/2, and STAT-induced genes)).

Cells identify pathogen invasion due to the presence of pathogen-associated molecular patterns (PAMPs) contained in viral components, which are recognized by host pattern recognition receptors (PRRs) [62]. Examples of viral PAMPs include some envelope or capsid proteins, viral nucleic acid, or intermediates of genome replication [62]. Upon PAMP engagement of a PRR, a signaling cascade is initiated that relies on post-translational modifications for proper coordination. These modifications include ubiquitination and phosphorylation, which facilitate the assembly of adaptor and enzymatic molecules needed to activate and inactivate transcription factors and other effector molecules [2,62,63]. These transitions in the transcriptional profile and functional proteome enable the cell to respond optimally to the pathogen and to communicate (e.g., cytokine secretion) with neighboring cells to limit viral replication and promote clearance. Examples of pathways critical in response to viral infection include IFN induction and signaling and NF-κB activation [64]. TRIM E3 ligases regulate IFN production and signaling as well as NF-κB induction at multiple levels, from PRR-mediated PAMP recognition to regulation of transcription factors and from promotion of signaling complex assembly to degradation of inhibitors [2,7]. In this section, we discuss TRIM regulation of antiviral pathways (summarized in Figure 1 and Figure 2).

### 2.2. TRIMs and the Retinoic Acid-Inducible Gene I (RIG-I)-like Receptor Pathway

Viral double-stranded RNA (dsRNA) or single-stranded RNA (ssRNA) containing 5’-triphophates produced during virus replication, in the cytoplasm of a host cell, act as a Retinoic Acid-Inducible Gene I RIG-I-like receptor (RLR) agonist [65,66,67,68]. RIG-I and melanoma differentiation-associated protein (MDA5), encoded by *Ddx58* and *Ifih1*, respectively, bind distinct viral RNA agonists yet they induce similar downstream antiviral pathways [69]. RLRs are ATP-dependent RNA helicases that have two N-terminal caspase-activated recruitment domains (CARDs), a central DEAD box, and an auto-inhibitory C-terminal domain [70]. The unique PAMPs recognized by these receptors enable the host to respond to a broader range of pathogens [69]. Upon engagement of the PAMP with the RLR, a conformational shift exposes the CARDs, which allows homo-oligomerization and recruitment of the RLRs to their adaptor mitochondrial antiviral signaling protein (MAVS) at the mitochondrial outer membrane (MOM) [66,71,72,73]. A variety of factors influence the activation of RLRs downstream of PAMP recognition including ATP hydrolysis [66,74], RLR oligomerization [71,72,74], and post-translational modifications [75,76,77,78,79]. Interaction of the N-terminal CARDs of both the RLRs and MAVS induces the adaptor to form prion-like aggregates and exposes domains to recruit critical RING E3 ligases including tumor necrosis factor (TNF) receptor-associated factors (TRAFs) 3 and 6 [80,81].

Downstream of TRAF6, the UBD-containing adaptor TGF-β-activated kinase 1(TAK1)/Mitogen activating protein 3K7 (MAP3K7)-binding protein (TAB) 2/3 recruits the critical kinase TAK1 [82,83,84]. TAK1 auto-phosphorylates to enable the phosphorylation of NF-κB essential modulator (NEMO), the regulatory domain of inhibitor of NF-κB (IκB) kinase (IKK) complex, to activate the enzymatic domains of IKKα and β [83,85]. IKKα and β then phosphorylate IκB, resulting in the recruitment of another E3 ligase, β-TrcP (β-Transducin Repeat Containing E3 Ubiquitin Protein Ligase). β-TrcP ligates K48-linked ubiquitin to IκB, inducing the proteasome-mediated degradation of the NF-κB inhibitor [63]. Once the inhibitor is destroyed, NF-κB is phosphorylated and its nuclear localization sequence is exposed allowing nuclear translocation [63,86]. Inside the nucleus, NF-κB regulates the transcription of a variety of genes including pro-inflammatory cytokines and chemokines, such as pro-IL-1β, TNF-α, and IL-6, and negative regulators of the pathway to limit an exacerbated inflammatory response [86].

In addition to activation of NF-κB, RLR signaling induces type-I IFN [73]. TRAF3, in cooperation with NEMO, recruits and stabilizes TRAF family member-associated NF-κB activator (TANK) or nucleosome assembly protein (NAP1) which are critical in linking TANK binding kinase 1 (TBK1), and in some cases inhibitor of kappa light polypeptide gene enhancer in B cells (IKKε), to the MAVS signalosome [81,82]. Once activated, TBK1 and/or IKKε phosphorylate the IFN regulatory factor (IRF) 3 and IRF7 [87,88]. Upon phosphorylation, the IRFs homodimerize and translocate to the nucleus where they bind to DNA regulatory regions [87,89]. To induce optimal IFN-β transcription, activated IRF3, NF-κB, and AP-1 (activator protein 1) must translocate to the nucleus and bind to their respective regulatory regions of the *Ifnb1* promoter [64]. The resulting IFN-β is then secreted and signals in a paracrine and autocrine manner. Binding of IFN-β to its heterodimeric receptor results in the activation of tyrosine kinases, Janus kinase 1 (JAK1) and tyrosine kinase 2 (Tyk2), which phosphorylate signal transducer and activator of transcription (STAT) 1 and STAT2. Following phosphorylation, STAT1 and STAT2 heterodimerize and associate with IRF9 to form IFN-stimulated gene factor 3 (ISGF3) and translocate to the nucleus [87]. Within the nucleus, ISGF3 binds to genes with an IFN stimulated response element (ISRE) in their promoter to activate transcription [87]. The resulting proteins expressed from these ISGs, such as PKR (protein kinase R), MxA (myxovirus resistance gene A), ISG15, and TRIMs, are involved in creating a cellular environment prohibitive to viral entry and replication [87]. As with other immune pathways, ISGF3 also promotes the transcription of type-I IFN negative regulators to mitigate deleterious effects [87].

TRIMs play a critical role in both the positive and negative regulation of the RLR pathway to ensure optimal virus restriction while minimizing self-inflicted damage (Figure 1). Several TRIMs have been shown to positively regulate the receptors RIG-I and MDA5 [90,91,92]. The best characterized example of TRIM-mediated RIG-I activation involves TRIM25. TRIM25 ligates K63-linked poly-Ub chains onto the N-terminal CARD at K172, which induces downstream signaling [92]. Additionally, TRIM25 catalyzes the synthesis of unanchored K63-linked poly-Ub chains, which facilitate RIG-I oligomerization and stabilization [77]. Both oligomerization and stabilization of RIG-I promotes the interaction of its CARDs with MAVS [93]. Adding complexity to this interaction, TRIM25 K48-linked polyubiquitination negatively regulates RLR activation, but the ubiquitin specific protease 15 (USP15) can specifically disassemble these poly-Ub chains to stabilize TRIM25 [94]. TRIMs 4, 13, and 38 have also been implicated in positive regulation of the RIG-I pathway. Similar to TRIM25, TRIM4 also catalyzes the ligation of K63-linked poly-Ub chains onto RIG-I CARD [91]. Additionally, TRIM38 functions as an E3 SUMO (small ubiquitin-like modifier) ligase and SUMOylates both RIG-I and MDA5 to prevent the ligation of K48-linked poly-Ub chains thus stabilizing these PRRs [7,95,96]. The capacity of multiple TRIMs to activate RIG-I suggests that ubiquitination is crucial in RIG-I signaling, but the relative contribution of each TRIM is not well understood. Perhaps multiple TRIMs allow for redundancy in the instance that one TRIM is inhibited or if TRIMs play cell-type specific roles in RLR signaling.

Recently TRIM65 was identified as an E3 ligase of MDA5. Unlike TRIM25, TRIM65 ubiquitinates MDA5 at the RNA helicase domain [90]. The covalent linkage of K63-linked poly-Ub onto K743 promotes MDA5 oligomerization and downstream activation of IRF3 [90]. Demonstrating the specificity of MDA5 activation, TRIM65 only promotes the restriction of encephalomyocarditis virus (EMCV), a picornavirus, and not vesicular stomatitis virus (VSV), a rhabdovirus [90]. In mouse cells, TRIM13 was shown to impair MDA5-mediated activation of the IFN pathway through an unclarified mechanism [50]. Another TRIM inhibitor of the MAVS pathway is TRIM59, which interacts with evolutionarily conserved signaling intermediate in Toll pathways (ECSIT) and MAVS and subsequently inhibits the transcription of IRF3 and NF-κB target genes [97]. Although TRIMs have not been identified as RIG-I negative regulators, their role in MDA5 inhibition suggests there may be unidentified TRIM-mediated RIG-I inhibition.

The role of TRIM25 in the regulation of RLR pathways and/or type-I IFN induction has been shown to be conserved among different species. In fact a diverse range of vertebrates encode RIG-regulating TRIMs. In salmonids, TRIM25, MAVS, MDA5, and RIG-I were induced following infection with an alphavirus although the signaling pathways were not addressed directly [98]. Duck TRIM25 catalyzes the synthesis of unanchored poly-Ub chains to activate RIG-I [99]. Despite lacking lysine 172 in duck RIG-I, duck TRIM25 ubiquitinates RIG-I’s CARD domains and promotes RLR signaling [99]. In chicken cells, despite lacking a functional RIG-I gene, knockdown of chicken TRIM25 results in reduced IFN-β upon infection with specific strains of the influenza A virus (IAV) [100], suggesting that TRIM25 is involved in activation of IFN signaling through a RIG-I-independent mechanism, perhaps activation of MDA5 or MAVS. Expression of chicken TRIM25 is induced after Newcastle disease virus (NDV), poly(I:C) treatment, or poly(dA:dT) treatment [101], probably via a type-I IFN signaling-dependent pathway. Similar to human TRIM25, TRIM27-L stimulates IFN-β production in response to IAV infection in ducks [102]. This anti-viral benefit is absent in chickens and turkeys, as they do not carry the TRIM27-L gene in their TRIM cluster [102]. However, expression of duck TRIM27-L and d2CARD in chicken DF1 cell lines was shown to facilitate IFN-β and MX1 expression [102]. This difference may account for the different pathologies in avian species as waterfowl are typically more resistant to some strains of IAV as compared to chickens.

Downstream of RLR activation, a variety of TRIMs promote MAVS signaling. TRIM25 has been implicated in the K48-linked polyubiquitination of MAVS, which results in its proteasome-mediated degradation and release of downstream signaling molecules (TBK1, NEMO, and possibly TRAF3) to induce type-I IFN production [103]. Recently, TRIM31 has been described to mediate the K63-linked ubiquitination of MAVS at lysines 10, 311, and 461 [104]. This ubiquitination promotes the prion-like aggregation of MAVS needed for optimal signaling, exemplified by the decrease in TBK1 and IKKε phosphorylation [104]. In a TRIM31-deficient murine model, TRIM31 knock-out mice demonstrated an increased susceptibility to VSV infection and an upregulation in IFN-β production. Although RIG-I was demonstrated to be required for activation of TRIM31 function, its role in MDA5-mediated signaling was not investigated in-depth [104]. TRIM14 has been demonstrated to play a crucial role in linking the NF-κB and IRF3 branches of RLR signaling [30]. Despite lacking a RING domain, TRIM14 interacts with NEMO via its PRY-SPRY domain and promotes K63-linked ubiquitination of NEMO, which is critical in recruiting NEMO to MAVS [30]. The role of TRIM44 stabilization of MAVS has also been characterized [7,105]. Although no TRIM inhibitors of MAVS have been described, screens of TRIMs that inhibit MAVS-mediated type-I IFN production may reveal such TRIMs.

In addition to regulating RLRs and MAVS, TRIMs modulate downstream adaptors and enzymes. Several of these TRIMs can likewise mitigate signaling downstream of other PRRs that converge on shared molecules such as NEMO and TAK1, but TRIMs identified to be involved at the level of signaling using RLR induction are described below. Recently, the short isoform of TRIM9 (TRIM9s) was shown to bridge GSK3β ( Glycogen synthase kinase 3 beta) to phosphorylated TBK1 to promote TBK1 oligomerization and activation of IRF3 [106]. To facilitate the interaction between GSKβ and pTBK1, TRIM9s must be auto-ubiquitinated [106]. This activation of TBK1-signaling occurs downstream of RLR and STING signaling [107] and biases the immune response toward the type-I IFN pathway while limiting NF-κB-induced transcription [106]. TRIM11’s coiled-coil domain interacts with the coiled-coil domain 2 of TBK1 to prohibit the kinase’s interaction with adaptors NAP1 or TANK [108]. Impairing this interaction results in lack of IFN-β production [108]. NAP1 is targeted for degradation following TRIM38-mediated K48-linked poly-Ub, which likewise decreases activation of IFN-β [109]. Interaction between the ARF (ADP-ribosylation factor domain) (C-terminus) domain of TRIM23, and both the coiled-coil 1 and LZ domains of NEMO, allow TRIM23 to facilitate K27-linked ubiquitination of NEMO [110]. This ubiquitination of NEMO facilitates the activation of IRF3 and NF-κB signaling downstream of pathogen recognition, but not TNF-α signaling [110]. Similar to the described TRIM9s mechanism, TRIM26 is able to interact with TBK1 and auto-phosphorylates K27-linked poly-Ub chains to bridge TBK1 and NEMO [111]. This interaction was demonstrated downstream of MAVS signaling and promoted IRF3 activation [111]. Indirectly, TRIM68 antagonizes IFN-β transcription [112]. This TRIM is able to inhibit both toll-like receptor (TLR) and RLR-driven activation of the type-I IFN pathway [112].

Several TRIMs are likewise implicated in the inhibition of the NF-κB activation branch of PRR signaling. Murine specific TRIM30α negatively regulates the TAB2/TAB3 complex, which impedes the recruitment and activation of TAK1, thus preventing the phosphorylation of NEMO and subsequent NF-κB activation [7,113]. Through a different mechanism in the brain, the long isoform of TRIM9 (TRIM9L) inhibits β-TrcP [33]. Perturbation of β-TrcP inhibits both canonical and non-canonical NF-κB activation [33]. The TRIM9L protein sequence includes a degron motif that, when phosphorylated at serine residues 76 and 80, recruits β-TrcP [33]. Titrating β-TrcP from the NF-κB inhibitors prevents their degradation and subsequent pro-inflammatory signaling [33]. Several other TRIMs also inhibit NF-κB activation including TRIM11 [114], TRIM29 [115], and TRIM39 [116]. The mechanisms for TRIM11 regulation have not been clearly elucidated, but TRIM29 targets NEMO for degradation in alveolar macrophages [115] and TRIM39 stabilizes Cactin [116], a nuclear, negative regulator of NF-κB. The bias of TRIMs in the negative regulation of the NF-κB branch of RLR signaling suggests that TRIMs may play a role in promoting the type-I IFN pathway at the cost of NF-κB activation, and further supports the hypothesis that groups of TRIMs may have evolved as part of the antiviral type-I IFN system [48]. However, it is important to note that some studies showing negative regulatory roles of TRIMs have only used overexpression assays with large concentrations of TRIM expressing vectors, which could lead to artifacts. However, it is now clear that overall TRIMs act at several levels to regulate RLR signaling to balance viral clearance and cell survival.

### 2.3. TRIMs and STING Signaling

In addition to regulating cytosolic RNA-stimulated responses, TRIMs also regulate the pathways following cytosolic DNA recognition. In the cytoplasm, the host expresses multiple DNA and RNA receptors aside from RLRs, including IFI16 (Interferon Gamma Inducible Protein 16), cyclic GMP-AMP synthase (cGAS), and DDX41. The listed double-strand (ds) DNA receptors activate the adaptor molecule stimulator of IFN genes (STING) at the endoplasmic reticulum (ER) [117,118,119,120,121]. Upon recognition of dsDNA, which can result from infection with DNA viruses, cGAS oligomerizes and catalyzes cyclic dinucleotide (c-GMP-AMP) synthesis [95]. c-GMP-AMP and other dinucleotides activate STING and induce STING dimerization [95,119,120]. Consequently, STING recruits TBK1, which phosphorylates IRF3 for type-I IFN induction [117]. The DNA helicase DDX41 recognizes both cytoplasmic dsDNA and cyclic dinucleotides, both of which promote DDX41 activation of STING [121,122].

Five TRIMs are known to influence STING-mediated signaling (Figure 1). The PRY-SPRY domain of TRIM21 interacts with DDX41’s helicase domain and catalyzes ubiquitination at lysine residues 9 and 115, targeting the PRR for degradation [123]. This degradation pathway occurs in myeloid dendritic cells and restricts type-I IFN induction [123]. The murine-specific TRIM, TRIM30α, ubiquitinates STING following herpes simplex virus 1 (HSV-1) and targets the adaptor protein for degradation [124]. Both TRIM32 and TRIM56 facilitate K63-linked polyubiquitination of STING to promote dimerization and activation of STING-mediated antiviral responses [125,126]. Finally, TRIM38 SUMOylates cGAS and STING similar to the SUMOylation of RIG-I and MDA5 [95]. SUMOylation inhibits the ligation of K48-linked ubiquitin and results in their stabilization [95].

### 2.4. TRIMs and TLR Signaling

In addition to regulating cytosolic PRR signaling, TRIMs also modulate membrane-bound PRRs including toll-like receptors (TLRs) (Figure 1). Several TLR family members recognize viral PAMPs. The main TLRs involved in virus recognition include endosome-localized TLRs 3, 7, 8, and 9 [127]. TLR3 recognizes both double-stranded RNA and the viral RNA mimic poly(I:C), TLR7 and TLR8 recognize single-stranded RNA, and TLR9 recognizes CpG [127]. TLR4 is associated mainly with the plasma membrane, although it can also be internalized in endosomes, and can recognize some viral surface antigens. TLRs 4, 7, 8, and 9 signal through IRAKs (Interleukin-1 receptor-associated kinase) 1 and 4, which are recruited via the adaptor molecule MyD88 (Myeloid differentiation primary response gene 88) [127]. TRAF6 then re-localizes to the TLR signaling complex to activate TAK1, inducing NF-κB [82,127] similar to the pathway described in the aforementioned RLR section. TLR3 and 4 also signal through IRAKs 1 and 4, and interact with the adaptor TRIF (TIR-domain-containing adapter-inducing interferon-β), resulting in the activation of TRAF3. Ubiquitination downstream of TRAF3 induces TBK1- and IKKε-mediated phosphorylation of IRF3 and IRF7 to induce type-I IFN [127]. TRIM21’s PRY-SPRY domain interacts with IRFs, including IRF3, 5, and 7, to induce their degradation [128,129,130]. Although TRIM21 may promote degradation of IRFs downstream of other PRRs, the interaction between the two proteins may depend on the induction of a specific TLR pathway. TRIM38 targets TLR signaling at multiple points. Downstream of TLR2, 3, 4, or 7, TRIM38 targets TRAF6 for proteasome-mediated degradation following K48-linked polyubiquitination [131]. Downstream of TLR3 and TLR4 signaling, TRIM38 also targets TRIF and NAP1 for degradation [109,132]. Finally, TRIM56 has been shown to promote TLR3 activation via interaction with TRIF in a RING ligase-independent manner [133]. Perhaps this interaction promotes the stability of TRIF to facilitate downstream signaling. Disruption of adaptor protein availability thus bottlenecks antiviral signaling.

### 2.5. TRIMs and the Nucleotide-Binding Domain and Leucine-Rich Repeat-Containing Receptors (NLR) Pathway

Nucleotide-binding domain and leucine-rich repeat-containing receptors (NLRs) are another class of cytosolic receptors that recognize both PAMPs and damage-associated molecular patterns (DAMPs). DAMPs are host-derived molecules that are expressed only after a cell experiences stress and/or damage commonly due to inflammation [134,135]. Upon activation of a NLR, the receptor assembles an inflammasome in cooperation with the adaptor ASC to recruit pro-caspases [134,135]. The NLRP3 (NLR Family Pyrin Domain Containing 3) -induced inflammasome promotes the cleavage of pro-caspase 1 to caspase 1, which then promotes a pro-apoptotic response involving the caspase-1-mediated cleavage of pro-IL-1β and pro-IL-18 to their active forms IL-1β and IL-18 [134,135]. The secreted pro-inflammatory cytokines then promote further inflammatory responses, such as pyroptosis, which may be damaging to the host when uncontrolled [134,135]. In some instances another NLR, NOD2, may function as a cytosolic dsRNA receptor and converge with the RLR pathway at the level of MAVS [136,137].

At this point only a few TRIMs have been described to interact with NLRs in the control of viral infections. TRIM33 binds to and ubiquitinates DHX33, a cytosolic dsRNA receptor that acts upstream of NLRP3, at lysine K218 to facilitate inflammasome activation [138]. The knockdown of TRIM33 diminishes the activation of caspase 1 and likewise decreases the release of IL-1β and IL-18 [138]. The interaction of TRIM33 with DHX33 relies on the B-box and coiled-coil domains [138]. In contrast, the murine-specific TRIM30α impairs the NLRP3 inflammasome through an unknown mechanism [139]. Another NLRP3 inhibitor identified using a dextran sodium sulfate-induced colitis model is TRIM31 [140]. The coiled-coil domain of TRIM31 interacts with the leucine rich and NACHT domains of NLRP3 and ligates K48-linked poly-Ub chains to target NLRP3 for proteasome-mediated degradation [140]. Although the authors did not evaluate virus infection directly, *Trim31*-deficient cells stimulated with poly(I:C) express higher levels of NLRP3 compared to wild-type cells suggesting TRIM31 may regulate NLRP3 downstream of virus recognition [140]. TRIM27 is able to promote degradation of NOD2 [141], which may prohibit this receptor from recognizing DNA and RNA virus infection [136,137]. The further investigation of TRIM interactions with NLRs is important for understanding the immune response during bacteria-virus co-infections.

### 2.6. TRIMs and Cytokine Signaling

Several TRIMs are also involved in regulating the cytokine signaling following initial pathogen recognition (Figure 2). Downstream of TNF-α engagement with its receptor TNFR1, the TAK1 complex is recruited to activate NF-κB [63]. TRIM8 specifically promotes the activation of TNF-α-induced NF-κB signaling through inhibition of the NF-κB nuclear repressor protein inhibitor of activated STAT (PIAS) 3 [142]. The TRIM8-mediated repression of PIAS likewise promotes IL-6-dependent activation of STAT3 [143]. Specifically downstream of IL-1β and TNF-α signaling in IFN-β-primed cells, TRIM38 targets TAB2 for lysosomal degradation [49,144]. As described above, the degradation of TAB2 inhibits the recruitment and activation of TAK1 which blocks pro-inflammatory signaling. Zheng and colleagues showed that TRIM27 catalyzes K48-linked poly-Ub at residues K251 and K372 of TBK1 to promote proteasome-mediated degradation downstream of IFN signaling [145]. TRIM27-TBK1 interactions require the coiled-coil and B-box TRIM domains [145]. In endothelial cells, TRIM28 sustains the expression of TNFR1 and TNFR2 to promote the activation of pro-inflammatory pathways [146]. The role of TRIM28 in the activation of the endothelium may play an important, unexplored role in immune cell trafficking in response to infection.

In response to cytokines (i.e., IL-12) secreted from activated dendritic cells (DCs), IFN-γ (type-II IFNs) is released from T cells and natural killer cells [147]. After this, type-II IFN binds its receptor, and the downstream kinases JAK1 and JAK2 promote STAT1 phosphorylation and homodimerization to form gamma activating factor, which translocates to the nucleus to bind gamma-stimulated elements in gene promoters [147]. TRIM24 inhibits STAT1 transcription via binding to the STAT1 promoter [148]. In contrast, TRIM8 is able to destabilize SOCS-1 (Suppressor Of Cytokine Signaling 1), a negative regulator of the IFN-γ signaling pathway, resulting in increased type-II IFN signaling [149]. The regulation downstream of IFN signaling suggests that this is a negative regulatory mechanism to prevent inflammatory response overactivation. In response to type-I IFNs, TRIM6 catalyzes the formation of K48-linked unanchored poly-Ub chains to facilitate the activation of IKKε, which favors ISGF3 formation due to STAT1 phosphorylation at S708 [11]. This modification increases STAT1-STAT2 dimerization [150,151]. This TRIM6-enahnced activation of IKKε may also play a role in activating IFN-β transcription and translation downstream of RLR activation [11]. TRIM22 induces the lysosome-mediated destruction of FOXO4 and consequently impairs the transcription of IFN-β downstream of TLR3 and RLR signaling [152].

### 2.7. TRIMs and Adaptive Immunity

After the innate response to pathogen invasion, the host evolves an adaptive immune response. Establishment of an adaptive response requires the presentation of pathogen antigens to T and B lymphocytes. Although TRIM proteins have been mostly studied as regulators of innate immune responses and the role of TRIMs in the regulation of antigen presentation has not been investigated intensively, several studies indicate that TRIMs play an important role in regulating T cell activation. Expression of TRIM22, for example, has been shown to be down-regulated upon CD28/CD2-mediated activation despite being expressed at high levels in resting T cells [153]. In contrast, TRIM22 expression in T cells is increased following IL-2 and IL-15 cytokine signaling [154], both of which act as pro-survival signals. Recently, TRIM24 was shown to influence the expression of Th2-type cytokines but not other CD4^+^ T cell sub-types [155]. As Th2 cells predominantly act in allergy- and parasite-induced responses while Th1 cells are more important for viral clearance, deregulation of TRIM24 expression may impact the CD4^+^ population composition and the capacity of the host to efficiently clear the virus. In the past, TRIM27 was described as impairing the activation of CD4^+^ T cells via K48-linked poly-Ub of PI3KC2B (Phosphatidylinositol 4-phosphate 3-kinase C2 domain-containing subunit beta) [156]. This impairment was observed specifically in CD4^+^ T cells downstream of T cell receptor (TCR) engagement and not CD8^+^ T cells [156]. Additionally, TRIM28 depletion in vivo promotes expansion of the Th17 population resulting in an autoimmune phenotype [34]. Following TCR-mediated activation, TRIM28 was phosphorylated suggesting it plays a role T cell activation [34]. Throughout the lifespan of *Trim30* knockout mice, the ratio of CD4^+^ to CD8^+^ progressively increased and the CD4^+^ T cells proliferated abnormally [157]. The role of TRIM30 is intrinsic to CD4^+^ T cells, because the same defect was observed upon adoptive transfer of knockout CD4^+^ T cells to wild-type mice [157]. It remains to be seen whether other TRIMs play important roles in adaptive immunity. It is likely that TRIMs have unexplored functions in recognition of antigen presentation by the T cell receptor, and or in differentiation of CD4^+^/CD8^+^ T cells.

## 3. TRIM-Mediated Virus Inhibition

Despite the numerous host evasion mechanisms pathogens employ, a variety of host encoded molecules, such as TRIMs, are able to restrict viruses [2]. In addition to conferring an antiviral state indirectly by regulating cytokine production downstream of PRR signaling, TRIMs are capable of restricting the effectiveness of pathogens through direct interactions with viral proteins crucial to their entry, dissemination, or life cycle [6]. The categories of viral restriction include: inhibition of viral transcription, replication or translation, degradation or interference of viral proteins, and impairment of virus entry or exit. We next outline the various means by which hosts deploy TRIMs to counter and clear pathogens.

### 3.1. TRIMs and Retroviruses

#### 3.1.1. TRIM5α and Retroviruses

**Figure 3:**
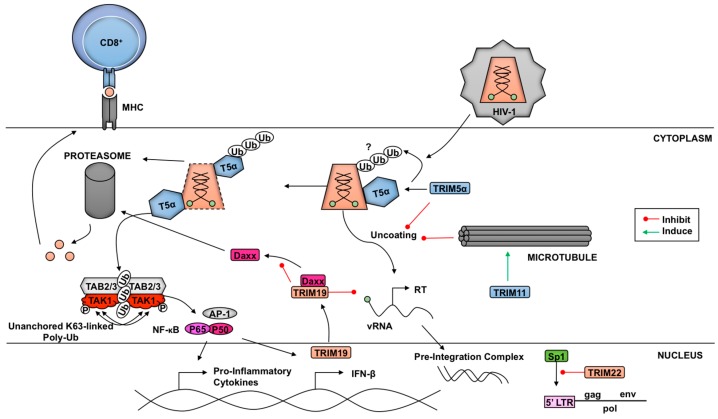
The role of TRIMs in retrovirus replication. TRIM5α oligomerization into a hexagonal lattice associates directly with HIV-1 capsids to promote premature uncoating. Recognition of HIV-1 capsid by TRIM5α also triggers NF-κB/AP-1-mediated innate immune signaling via synthesis of unanchored K63-linked poly-Ub chains that activate the TAK1 kinase. One potential mechanism of TRIM5α-mediated restriction could be involved ubiquitination of TRIM5α and proteasomal degradation of TRIM5α-capsid complexes. TRIM11 mobilizes cellular microtubule formation to prematurely uncoat HIV-1 and facilitate rapid release of the vRNA from the viral core, resulting in inhibition of virus replication. TRIM19 translocates to the cytoplasm and binds Daxx to prevent its degradation by the proteasome, allowing for Daxx-mediated disruption of HIV-1 reverse transcription (RT). TRIM22 inhibits Sp1, preventing LTR-mediated transcription. The connection between TRIM5α-mediated restriction and proteasomal-mediated degradation of HIV-1 requires further characterization. However, one potential model may involve improved proliferation of HIV-1-specific CD8^+^ T cells due to enhanced production of viral peptides from proteasome-mediated degradation of HIV-1 capsids.

TRIM5α is an example of a TRIM functioning as both a direct virus restriction factor as well as a pathogen-recognition receptor (Figure 3). In old-world monkeys, such as African green monkeys and rhesus macaques, TRIM5α enables natural resistance to HIV-1 while new-world monkeys, like owl monkeys, are protected from HIV-1 by fusion of TRIM5 with cyclophilin A (TCypA) [158,159,160,161]. Progress on the topic ranges from revealing the α isoform of TRIM5 as the restriction factor to discerning the role in restriction of each structural motif [158,159].

Several components inherent to TRIM5α play a role in HIV-1 capsid restriction, including dimerization, oligomerization, and ubiquitination [162,163,164]. There have been different models proposed for the mechanism of restriction and all have been attributed to the interaction of the SPRY domain of TRIM5α with the retrovirus capsid. Binding of TRIM5α to viral cores serves to prematurely uncoat the viral particle and induces early release of the viral genome. Elucidation of the TRIM5α higher order structure has revealed the significant contributions made by each component of the TRIM5α protein [26,163,164,165,166]. Monomeric TRIM5α can dimerize in an antiparallel fashion through the coiled-coil domains, thereby generating the most fundamental component needed to bind the viral capsid [163,167]. Once bound to its target, TRIM5α can form higher-order structures resembling a hexagonal net [164,165,168,169]. TRIM5α and TRIM5 variants from several primate species, including rhesus macaques, African green monkeys, and owl monkeys, are capable of forming flexible, hexagonal frameworks encompassing HIV-1 capsid surfaces [164]. The hexagonal nets are formed from TRIM5α trimers and are dependent on the TRIM5α’s B-box domain [165,166]. These trimers have been shown to be flexible and may allow for ideal binding of the SPRY domains on the highly variable HIV-1 capsid [165,166]. The ability of rhesus macaque TRIM5α (rhTRIM5α) to form higher-order structures upon binding to the capsid also allows the formation of TRIM5α RING dimers, which enhances its E3-ubiquitin ligase activity and innate anti-HIV-1 activity [29]. As one potential mechanism of rhTRIM5α-mediated restriction it was proposed that as the linker 2 regions of the dimer change their conformation, the SPRY domains that are bound to the HIV-1 capsid in their hexagonal lattice formation disturb the viral structure [163]. The induction of this conformational change disrupts the integrity of the viral core and may be responsible for pre-mature viral uncoating.

Proteasomal-mediated degradation of viral components is a host restriction strategy characteristic of many TRIM family proteins involved in host innate immunity. However, the functional role of the proteasome in TRIM5α-mediated HIV-1 restriction has been difficult to discern and has been subject of debate. Early work with proteasomal inhibitors, like MG132, suggested a two phase restriction of HIV-1 by TRIM5α. In the proteasome-independent phase, TRIM5α inhibits nuclear entry of the reverse transcription (RT) products [170,171]. In the second phase, TRIM5α can inhibit late RT products in the presence of a functional proteasome, suggesting TRIM5α may utilize the proteasome to disrupt the viral RT complex [170,171]. While proteasome inhibition can block disassembly and/or degradation of viral core components, it does not appear to rescue infectivity, which has led some investigators to conclude that degradation of capsid by the proteasome is not the mechanism of TRIM5α-mediated restriction [172]. In addition, thus far no study has described ubiquitination sites on the HIV-1 capsids, which could potentially link degradation of the capsid with proteasomal function. However, additional studies have shown proteasomal involvement in TRIM5α restriction of HIV-1 by degrading TRIM5α itself [173]. TRIM5α’s E3 ligase activity can facilitate auto-ubiquitination, thereby signaling for its own destruction by the host proteasomal machinery [36,173]. These occurrences have led some to speculate on a potential model where TRIM5α interacts with the HIV-1 capsid leading to auto-ubiquitination, capsid uncoating, and delivery to the proteasome [173,174,175,176]. Despite numerous investigations centered around proteasomal involvement, controversy within the literature surrounding the exact model connecting TRIM5α, HIV-1 cores, and proteasomes remains, and proteasome-dependent and independent mechanism have been proposed [175,176].

Polyubiquitination through the E3 ligase activity of TRIM5α’s RING domain may also be a factor involved in inhibition of retroviral replication [177]. In addition to its restriction activity, TRIM5α can induce antiviral type-I IFNs. Multiple TRIM5 orthologs induce AP-1-mediated innate immune signaling [169,178]. In the presence of the HIV-1 capsid, the E3 ligase activity of TRIM5 facilitates the generation of unanchored K63-linked poly-Ub chains. These K63-linked chains promote TAK1 autophosphorylation and activation, resulting in the induction of AP-1- and NFκB-mediated transcription [169]. Collectively, these recent findings serve to better characterize the mechanisms through which TRIM5α achieves inhibition of HIV-1 replication.

The restriction benefits conferred by TRIM5α can be observed in the progression to disease upon infection with simian immunodeficiency virus (SIV) in rhesus macaques. Course of infection studies with either a TRIM5α-sensitive or -resistant strain of SIV revealed that inoculation with a TRIM5α susceptible strain of SIV provided higher survival rates for rhesus macaques. Also, delayed development of the pathology associated with TRIM5α-sensitive SIV compared to subjects treated with a TRIM5α-resistant strain was observed [179]. In addition to these benefits, the restriction of the TRIM5α sensitive SIV strain correlated with prolonged maintenance of CD4^+^ central memory T cells [179]. The preservation of CD4^+^ central memory T cells has been extensively characterized as important in resisting viremia and generating CTL (cytotoxic T lymphocytes) subsets [180,181,182]. The prolonged presence of these CD4^+^ T cells in the TRIM5α-susceptible SIV-infected treatment may be responsible for the higher survival rate [179]. In spite of this, TRIM5α escape mutants were present [179]. Half of the subjects that received the TRIM5α-sensitive strain of SIV still developed AIDS, and a third succumbed to infection at a similar time as the TRIM5α-resistant group [179]. Sequencing of the SIV capsids from these macaques revealed two mutations in the region encoding the *gag* protein, which resulted in nonsynonymous substitutions that mimicked the alterations made by the authors to generate their TRIM5α resistant strain [179]. Subversion of TRIMs is a central evolutionary strategy for viral innate immune evasion, as demonstrated by the high mutational rate of retroviruses.

Although first described as a viral restriction factor against HIV-1 in old-world monkeys, humans and other species are capable of utilizing TRIM5α to inhibit other retroviruses. Human TRIM5α is incapable of restricting HIV-1, yet a single amino acid mutation in its PRY-SPRY domain can confer resistance to the pathogen [183]. Instead, human TRIM5α can bind to and restrict N tropic mouse leukemia virus (N-MLV) [183]. Interestingly, the 13-amino-acid stretch in the PRY-SPRY domain of primate TRIM5α is under strong positive selection [44]. TRIM5α paralogues have been identified in bovine [184,185], ovine [186], and piscine [40] species, and have also been shown to restrict retroviruses. Overall, TRIM5α plays a convergent role in the recognition and restriction of retroviruses.

Aside from its role in non-human primates, TRIM5α has been described functioning in a cell-type specific manner [187,188,189]. In contrast to conventional DC-SIGN^+^ (Dendritic Cell-Specific Intercellular adhesion molecule-3-Grabbing Non-integrin) DCs, Langerhans cells (LCs) benefit from the anti-retroviral capabilities of TRIM5α through an increased activity of the LC autophagocytic components [187]. Upon Langerin-mediated HIV-1 uptake, the presence of cellular autophagosomes increases as a result of TRIM5α-directed assembly, leading to targeting of the viral capsid for destruction [187]. Further evidence of cell-type specific HIV-1 restriction by TRIM5α was found in rhesus macaque DCs, which in contrast to macrophages appear to be permissive to HIV-1 infection [188]. The lack of TRIM5α-mediated restriction in both human and macaque conventional DCs may be due to SUMOylation of TRIM5α, which promotes its sequestration in nuclear bodies [189]. However, this lack of TRIM5α-mediated restriction in DCs provides an innate immune sensing advantage by allowing recognition of HIV-1 reversed transcribed DNA by cGAS, resulting in type-I IFN production [189]. It remains to be seen whether HIV-1 can actively promote or enhance SUMOylation of TRIM5α in DCs as mechanism to antagonize type-I IFN production.

The ability of TRIM5α to promote restriction of HIV-1 may go beyond direct intervention of the viral lifecycle. TRIM5α has also been linked to the activity of other components of cellular innate immunity including antigen presentation to cytotoxic T lymphocytes (CTL). TCypA and rhTRIM5α enhanced the ability of CD8^+^ T cells to identify HIV-1 infected cells, and promoted a HIV-1-specific immune response [174]. The presence of these TRIM5 orthologs was associated with increased associations between HIV-1 particles and the host proteasome [174]. It is speculated that this may facilitate improved HIV-1-specific CTL development, as amplified peptide concentrations could support enhanced antigen presentation to CD8^+^ T cells. The mechanism linking direct restriction of viral capsids with heightened CD8^+^ T cell activation warrants further investigation.

#### 3.1.2. Other TRIMs and Retroviruses

A plethora of TRIM-mediated restriction mechanisms targeting HIV-1 and other retroviruses have been proposed [15], and have been reviewed previously [6]. More recent reports acknowledged TRIM11 as a potent host restriction factor of HIV-1 [114,190]. The mechanisms of TRIM11-mediated restriction include curbing the amount of viral reverse transcription products allowed to accumulate in the host. Through an interaction with the viral capsid-nucleocapsid protein (CA-NC) complexes, TRIM11 promotes premature uncoating and release of the viral genetic material, reducing transduction efficiency. Neither proteasomal nor lysosomal inhibitor treatments recovered viral p24 protein in the pellets of TRIM11 overexpressing cells, suggesting that ubiquitin-mediated degradation by the proteasome or lysosomal acidification is not required for TRIM11-mediated uncoating [190]. Furthermore, while rhTRIM5α-mediated inhibition of HIV-1 is rescued by proteasome inhibitors [170,171], TRIM11-mediated inhibition was not, indicating that TRIM11 and TRIM5α restrict HIV-1 by different mechanisms [114,190]. In contrast, using the microtubule dynamics inhibitors nocodazole and taxol, the authors were able to demonstrate a restoration of HIV-1 capsid levels in cells supplemented with exogenous TRIM11, suggesting that microtubules may contribute to TRIM11-mediated HIV-1 restriction [190]. Earlier work with the use of nocodazole and taxol in a study examining HIV-1 and TRIM5α also demonstrated recovered HIV-1 infectivity in the absence of functional microtubules [191].

Although the exact mechanism of TRIM11-mediated restriction of HIV-1 in the aforementioned study has yet to be determined, the authors demonstrated that purified TRIM11 associated with in vitro assembled HIV-1 capsids [190], indicating that TRIM11 interacts directly with the capsid and it probably does not require TRIM5α or other cellular proteins for promoting untimely capsid uncoating. Microtubule involvement in viral uncoating has been implicated as both a host restriction and a viral propagation mechanism [192,193,194,195]. Functional microtubules and their associated motor proteins, like dynein, have been suggested as key components in genome release for both IAV and HIV-1 [192,193,194,195]. Interestingly, in the case of IAV, unanchored poly-Ub chains contained in the virion are recognized by the host histone deacetylase 6 (HDAC6), a component of the aggresome-autophagy pathway, which interacts with dynein and microtubules to promote viral uncoating [192,195]. It will be interesting to examine whether HIV-1 utilizes a similar mechanism and whether this is mediated by TRIM11. Additionally, several members of the TRIM family (TRIMs 1, 9, 18, 36, 46, and 67) have been shown to possesses a C-terminal domain motif allowing for association with cytoskeletal elements like microtubules [31]. So far only TRIM1 (also called MID2) and TRIM18 (also called MID1) have been shown to be directly involved in microtubule stabilization [196], and TRIM1 has been implicated in restriction of N-MLV [159]. Whether TRIM1-mediated restriction is dependent on microtubules, or whether other microtubule-interacting TRIMs may have viral restriction activity by microtubule-dependent mechanisms, remains to be seen.

TRIM-mediated suppression of HIV-1 replication has revealed additional avenues through which TRIMs subdue pathogens. TRIM22 prevents normal viral transcription events by regulating the effectiveness of the transcription factor Sp1 to bind the HIV-1 long terminal repeat (LTR) promoter region [197]. This restriction was independent of TRIM22’s E3 ligase activity and did not involve direct interaction between TRIM22 and Sp1, implying that the observed reduction in HIV-1 LTR-mediated transcription requires additional unspecified factors [197]. TRIM37 exhibits anti-retroviral functions through interference of HIV-1 infection, replication, and transcription, possibly interfering with DNA synthesis [198]. However further investigation into TRIM-mediated transcriptional inhibition will be required to reveal additional pathways in which TRIMs play a significant role.

Another example of indirect inhibition of virus replication by TRIMs is illustrated by the promyelocytic leukemia protein (PML)/TRIM19. PML/TRIM19 interferes with HIV-1 infection in human and murine fibroblasts through the reduction of reverse transcriptase products [199,200]. This effect was dependent on two events; the translocation of PML/TRIM19 from the nucleus to the cytoplasm in the presence of HIV-1 infection, as well as association of the PML/TRIM19 cytoplasmic bodies (CB) with Daxx [201]. PML/TRIM19 interacts with Daxx in a protective fashion in order to prevent its degradation by the proteasome, thereby making PML/TRIM19 a necessary component for Daxx-mediated inhibition of HIV-1 RT [201]. The inhibition of HIV-1 by PML/TRIM19 and Daxx may be cell-type dependent, since studies using different cell types have shown different results [200,201].

### 3.2. TRIM21 and Antibody-Dependent Intracellular Neutralization of Viruses

Another well characterized TRIM involved in pathogen recognition is TRIM21, which has been shown to detect intracellular antibody-opsonized viruses (reviewed previously in [6,7]). Some immunoglobulin-coated non-enveloped viruses are internalized into the host cell. Within most cells, a high affinity antibody receptor, TRIM21, binds to the highly conserved Fc region of virion-bound immunoglobulin (Ig)G, IgM, or IgA [7,202], and targets the virus for degradation by the proteasome before the virus can transcribe its genes [203]. TRIM21’s PRY-SPRY domain interacts with Fc residues conserved across mammalian species [204]. TRIM21 binding with an immunoglobulin-virion (Ig-V) complex triggers tightly regulated intracellular antibody neutralization and pro-inflammatory pathways [7,202]. Upon engagement of TRIM21 with the Ig-V complex within the cytoplasm, the E2 Ube2W (Ubiquitin Conjugating Enzyme E2 W) monoubiquitinates TRIM21, which promotes Ube2N/Ube2V2 E2 complex recruitment to TRIM21 for K63-linked poly-Ub [12]. Following poly-Ub, TRIM21 is recruited to the proteasome to initiate degradation of the antibody-bound virus. Concurrently, with proteasome-mediated degradation, Poh1 de-ubiquitinates TRIM21 [12]. This antibody-dependent intracellular neutralization appears to be limited to non-enveloped DNA and RNA viruses that can enter the host cytosol with immunoglobulin attached to the virion surface [205]. The de-ubiquitinated TRIM21 is then able to promote both IFN induction and NF-κB activation [12,205,206], hypothesized to result from the release of the viral genome into the cytoplasm for PRR-mediated recognition [207]. However, some antiviral cytokines are induced independent of PRRs [207], but the pathway is not well characterized. In addition, it has also been proposed that recognition of the Ig-V complex in the cytoplasm by TRIM21 triggers the synthesis of unanchored K63-linked poly-Ub chains that activate NF-κB, AP-1 and IRF3 pathways [205]. Notably, when the affinity of TRIM21 for the Fc portion of an antibody is decreased, the viral neutralization efficiency is maintained while the activation of cytokine signaling is diminished [208]. This suggests that the association of TRIM21 with Ig-V complexes is important for triggering antiviral responses. TRIM21-deficient mice infected with mouse adenovirus 1 experienced lethal disease while wild-type mice were protected, exemplifying the importance of TRIM21 in viral neutralization in vivo [209]. Most likely, these mechanisms are shared in other species due to the highly conserved Fc and TRIM21 interacting residues, however the activity of TRIM21 as an intracellular antibody receptor in non-human, non-murine models has only been demonstrated in pig cells infected with foot-and-mouth disease virus [210].

### 3.3. TRIMs and Negative Sense RNA Viruses

**Figure 4:**
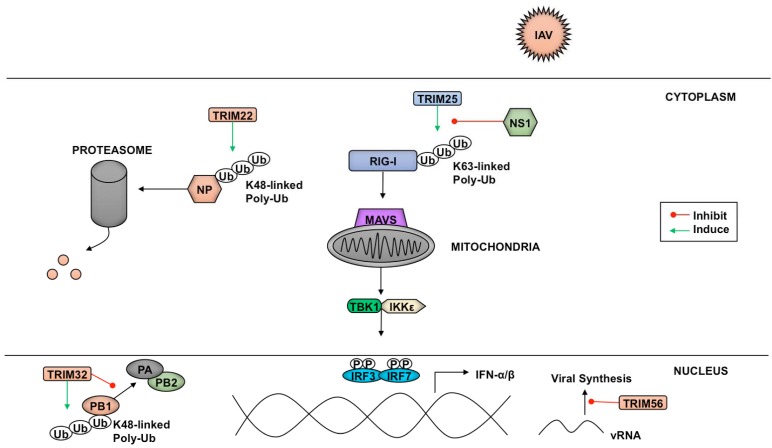
Effects of TRIMs on influenza virus components and IFN antagonism. The TRIM25-binding domain of the viral NS1 protein prevents TRIM25-mediated activation of RIG-I. TRIM56 has been proposed to target viral 5’ triphosphate RNA and inhibit vRNA synthesis. TRIM22 targets the viral nucleoprotein, while TRIM32 prevents association of PB1 to the viral polymerase complex through K48-linked polyubiquitin chains that target the viral components for proteasome-mediated degradation.

TRIM family proteins can target several components of pathogens ranging from structural elements to factors essential for transcription and replication. A variety of influenza A virus (IAV) and influenza B virus (IBV) proteins are targets of TRIM-mediated inhibition of virus replication (Figure 4). Despite being a target of IAV-NS1 (nonstructural protein 1)-dependent IFN antagonism, TRIM25 interacts with the N-terminus of IBV-NS1 preventing the viral protein’s C-terminal component from binding viral RNA [211]. Preventing IBV-NS1 from interacting with RIG-I allows signaling through this RLR pathway to proceed [211].

Another TRIM-mediated anti-IAV mechanism is polyubiquitination of IAV nucleoprotein (NP) by TRIM22, which leads to NP proteasome-mediated degradation and reduced virus replication [212]. The polymerase basic protein 1 (PB1) of IAV is also a target of TRIM-mediated inhibition. PB1 is one of the three components that make up the RNA-dependent RNA-polymerase responsible for transcribing the eight segments of the IAV genome [213]. TRIM32 facilitates K48-linked polyubiquitination of PB1, resulting in enhanced turnover of this viral subunit, and a reduction in viral titers [214].

Aside from targeting pathogen components for proteasomal degradation via ubiquitination, some members of the TRIM family have been shown to enact their restriction factor capabilities through other means. The TRIM56 C-terminal domain, rather than its E3 ligase activity, is required to reduce IAV and IBV replication. The mechanism TRIM56 employs to diminish vRNA levels of both influenza viruses is currently unknown, although inhibition of translation through direct interaction between TRIM56 and the vRNAs has been proposed [215].

### 3.4. TRIMs and Positive Sense RNA Viruses

**Figure 5:**
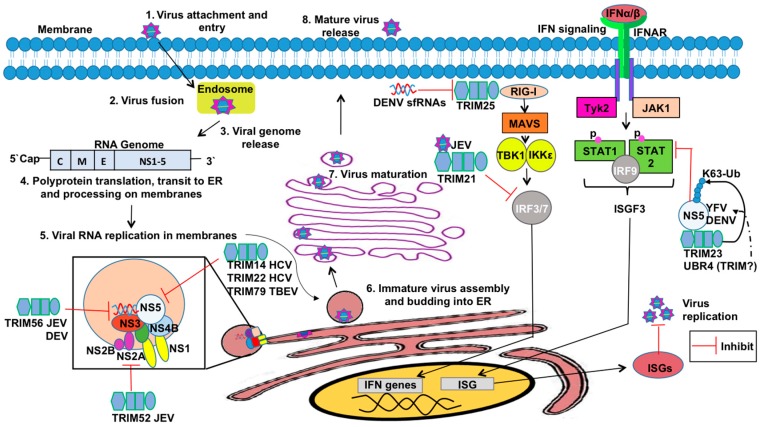
The roles of TRIMs in flavivirus infection and innate immune antagonism. Flaviviruses are internalized by receptor-mediated endocytosis and by clathrin-mediated endocytosis (1). Fusion of virus membrane with host endosomal membrane (2). RNA genome is released into the cytoplasm (3). The positive-sense genomic ssRNA is translated into a polyprotein, which is cleaved into all structural and non-structural proteins (4). Replication takes place at the surface of endoplasmic reticulum in cytoplasmic viral factories (5). In this step, the TRIMs restrict virus replication, degrading viral proteins such as NS2A in Japanese encephalitis virus (JEV) by TRIM52 and viral RNA inhibition in JEV and Dengue virus (DENV) by TRIM56. TRIM22 and TRIM79 degrade NS5 protein in hepatitis C virus (HCV) and tick-borne encephalitis virus (TBEV), respectively. Virus assembly occurs at the endoplasmic reticulum. The virion buds at the endoplasmic reticulum and is transported to the Golgi apparatus (6). The prM protein is cleaved in the Golgi, thereby maturing the virion, which is fusion competent (7). Release of new virions by exocytosis (8). TRIMs and antagonism function also are used by flaviviruses. TRIM21 inhibits IFN-β production during JEV infection. TRIM23 promotes yellow fever virus replication. DENV short noncoding sfRNAs bind TRIM25 to inhibit IFN expression.

TRIMs have also been reported to mediate restriction against Flaviviruses (Figure 5). The *Flavivirus* genus comprises more than 70 viruses including a number of important human pathogens such as Dengue virus (DENV), Zika virus (ZIKV), West Nile virus (WNV), tick-borne encephalitis virus (TBEV), Japanese encephalitis virus (JEV), hepatitis C virus (HCV), and yellow fever virus (YFV) [216]. Flaviviruses are small enveloped viruses hosting a positive-sense single-stranded RNA genome. Several flaviviral proteins are associated with viral persistence, immune system evasion, or viral replication [217].

HCV encodes a nonstructural protein, NS5A, which inhibits the phosphorylation and nuclear translocation of STAT1 in the IFN-α2-induced JAK/STAT pathway via their IFN sensitivity-determining region [218,219]. TRIM14’s SPRY domain specifically interacts with NS5 of HCV and induces NS5A degradation [220], which is an example of TRIM-mediated IFN-independent inhibition. TRIM22 specifically binds the NS5A-D1 protein (Domain 1) via its SPRY domain and utilizes its E3 ubiquitin ligase activity to target NS5A for removal [221].

Wenchun and colleagues have shown that TRIM52 interacts with the NS2A protein of JEV and targets the protein for proteasome-mediated destruction [222]. NS2A is a small, hydrophobic transmembrane protein involved in the virus life cycle and subversion of host antiviral responses [223,224], including inhibition of the double-stranded RNA-activated protein kinase PKR during JEV infection [225]. TRIM52-dependent inhibition of JEV NS2A protein occurs in BHK-21 and 293T cells and is therefore important for restricting JEV replication [222].

Recent evidence also suggests that the RING and C-terminal domains of TRIM56 may be important in the restriction of other Flaviviruses, including YFV and DENV [226], but the mechanism of action remains elusive. TRIM56 might modulate post-translational modification of one or more viral proteins and/or host factors to suppress viral replication [227]. Although TRIM56 fails to restrict HCV replication when overexpressed in human hepatoma Huh7 cells [228], TRIM56 overexpression in HEK293 cells support some selectable HCV RNA replicons at very low efficiencies [227]. Perhaps TRIM56 facilitates degradation of viral proteins similar to mechanisms observed with TRIMs 14 and 22 against HCV and TRIM52 against JEV. Further studies are needed to elucidate the underlying molecular mechanisms of TRIM56-mediated restriction of DENV and YFV. Interestingly, TRIM56 is capable of binding to the protease N^pro^ of bovine diarrheal virus (BVDV), a Pestivirus of the *Flaviviridae* family [228]. The restriction of this viral protein is critical considering that N^pro^ is capable of mediating IRF3 degradation, thus impairing production of IFN-β [228]. TRIM56-mediated restriction of BVDV is specific, as closely related viruses are not likewise impaired in their replication [228].

TRIM79α, also known as TRIM30-3 or TRIM30D, is present only in rodents. TRIM79α is highly expressed in the spleen, lymph node, and bone marrow in a type-I IFN-dependent manner, and is required for effective restriction of TBEV replication [229]. TRIM79α is an important mediator of the innate cellular response to restrict Langat virus (a member of the TBEV serogroup) infection by targeting the viral RNA polymerase and major IFN antagonist, NS5 [229]. NS5 has a methyltransferase and RNA-dependent RNA polymerase activity that associates with NS3 and NS2B to form the viral replication complex. NS5 inhibits IFN-α/β-dependent responses by preventing JAK-STAT signaling and thus suppresses IFN-stimulated gene (ISG) expression [229,230,231,232]. Taylor and colleagues demonstrated that TRIM79α interacts with NS5 from LGTV and TBEV and blocks the replication of these viruses via a lysosomal-targeting mechanism. Despite NS5 being the most conserved of the flaviviral proteins, TRIM79α did not target NS5 from WNV, nor could it inhibit WNV replication [232].

Besides Flaviviruses, other positive-sense RNA viruses have been reported to be inhibited by TRIM-mediated mechanisms and can occur through both direct and indirect means. For example, TRIM25 acts as a cofactor for the Zinc-finger antiviral protein (ZAP), a member of the poly(ADP-ribose) polymerase family that is known to bind and promote viral RNA degradation [233]. TRIM25 enhances ZAPs’ ability to inhibit Sindbis virus translation [233]. However, although TRIM25 is capable of promoting K48- and K63-linked polyubiquitination of ZAP isoforms, ubiquitination does not appear to affect ZAP antiviral activity [233]. It will be of interest to elucidate what other factors may be ubiquitinated by TRIM25 that could affect ZAP antiviral activity. TRIM19-IV has been shown to confer resistance to encephalomyocarditis virus (EMCV) hampering viral replication and protein synthesis [234]. This ability was shown to originate from TRIM19-IV’s C-terminus, specifically through an interaction with the viral 3D polymerase (3Dpol), leading to nuclear body sequestration of the viral polymerase [234]. Additionally, SUMOylation of TRIM19-IV was required to facilitate restriction of EMCV [234].

### 3.5. TRIMs and DNA Viruses

TRIMs play an additional role in restricting DNA virus replication. Eight different TRIM proteins (TRIM5, 6, 11, 14, 25, 26, 31, and 41) were identified to inhibit hepatitis B virus (HBV) [235]. In particular, TRIM41 was the only TRIM from this group of eight that specifically reduced both the enhancers I and II components of HBV. This inhibition of viral transcription required TRIM41’s RING and PRY/SPRY domains, implicating its E3 ligase activity [235]. TRIM22 is associated with HBV clearance in acutely infected chimpanzees, and possesses anti-HBV activity under physiological conditions [236]. Gao and colleagues also reported that TRIM22 was one of the most strongly induced TRIM family molecules in human hepatoma HepG2 cells after treatment with IFNs [237].

Epstein–Barr virus (EBV) replication and transcription activator (Rta) protein activates EBV lytic genes for proliferation [238]. TRIM5α promotes the ubiquitination of Rta upon interaction, thereby blocking the EBV lytic cycle [238]. The involvement of TRIM5α in a non-retroviral innate immune response implies TRIMs possess diverse, situation-specific functions that have yet to be characterized. TRIMs may also operate as recognition receptors for other components of host immunity. TRIM19 isoforms are able to capture varicella-zoster virus nucleoproteins to impede nuclear egress and thus block the release of new virions [239]. Comprehension of the multiple roles TRIM-family proteins play may become critical in discerning the impact that they have on the innate immune response.

## 4. Viral Antagonism of TRIMs

As we have described, TRIM family proteins play an important role in innate immunity and counter pathogens [7]. In retaliation to this evolutionary pressure, pathogens have adapted to antagonize TRIMs. Mechanisms of viral-mediated TRIMs restriction range from impairment of TRIM-promoted innate immune signaling complex assembly to direct hindrance of the TRIM proteins. Interfering with TRIMs, either directly or indirectly, undermines their intended function, and enables viruses to gain an early advantage over the host [58]. The following section highlights several recent examples that elucidate viral manipulation of TRIMs.

### 4.1. Antagonism of TRIMs by RNA Viruses

A broad range of RNA viruses have evolved effective evasion strategies to manipulate host antiviral immunity. Influenza viruses employ well-studied examples of TRIM antagonism (Figure 4). The nonstructural protein 1 (NS1) of influenza A and B virus (IAV/IBV) has been characterized as a viral antagonist of host innate immunity through interactions with TRIM25 [57,100,240,241]. As described above, TRIM25 plays a critical role in the activation of the RLR pathways [57,242,243]. The NS1 protein from IAV directly interacts with the coiled-coil domain of TRIM25 to impede its multimerization [57,244]. Since dimerization is required for TRIM25 E3 ligase activity, NS1 binding to TRIM25 leads to impaired ubiquitination of the RIG-I and downstream signaling, resulting in a reduced antiviral response [57,244].

The TRIM25 interaction with IAV NS1 is species-specific. Human TRIM25 interacts with IAV strains isolated from many species while chicken TRIM25 binds only NS1 from avian strains and murine TRIM25 did not bind any NS1 [100]. Riplet, a close relative of TRIM25, lacks a B-box domain but shares homologous RING and SPRY domains. Its predicted coiled-coil structure also binds NS1 to inhibit RIG-I signaling in mice and humans [100]. Aside from interacting with TRIM25, IAV NS1 also associates with host RIG-I directly [244]. As seen with IAV, the N-terminal domain of IBV NS1 is also able to block the Lys63-linked ubiquitination of RIG-I and subsequent antiviral signaling downstream of the RLR pathway [211].

Members of the *Coronaviridae*, *Flaviviridae*, and *Bunyaviridae* families also encode viral proteins that inhibit TRIM-mediated regulation of RLR signaling. Severe acute respiratory syndrome and Middle East respiratory syndrome coronavirus (SARS/MERS-CoV) are large, positive-sense single-stranded RNA viruses. Akin to the influenza virus NS1 protein, the nucleoprotein of SARS-CoV was demonstrated to interact with the SPRY domain of TRIM25, preventing the necessary interaction and subsequent ubiquitination of the RIG-I CARD domains [245]. A similar loss of RIG-I-induced IFN-β is achieved when MERS-CoV nucleoprotein associates with TRIM25 [245]. For DENV, Manokaran et al. compared two viral sequences (PR1 and PR-2B) and identified mutations that resulted in the increased production of subgenomic flavivirus non-coding RNAs (sfRNAs) by the PR-2B strain [55] (Figure 5). The PR-2B sfRNAs were capable of binding to host TRIM25 and prevented USP15-mediated deubiquitination [55], which is crucial for activation of RIG-I [94]. This data provides unique molecular insight into the epidemiological fitness of DENV, suggesting that DENV sfRNAs can bind to host proteins to promote viral evasion of innate immunity [55]. The NSs protein of severe fever with thrombocytopenia syndrome virus (SFTSV), a negative-sense RNA virus in the family *Bunyaviridae*, interacts directly with TRIM25, and indirectly with RIG-I and TBK1 to isolate these signaling molecules from associating with MAVS [246]. As with the other viral protein-TRIM25 interactions described above, downstream activation of IRF3 and subsequent IFN-β production are impaired [246]. TRIM25 is a common target of a diverse group of RNA viruses, suggesting that other pathogens may also impair TRIM25-mediated stimulation of the RLR pathway.

Similar to IAV, HIV-1 encodes multiple proteins that restrict TRIM function (Figure 3). For example, HIV-1 Vpr protein has been suggested to play a role in TRIM manipulation [114]. Interestingly, protein expression levels of TRIM11 have shown to be under the control of Vpr in a dose-dependent manner, where the presence of TRIM11 was decreased when the concentration of Vpr was low [114]. Currently, the mechanism HIV-1 Vpr employs to antagonize TRIM11 is unknown. Following infection of human neural precursor cells (hNPCs) with HIV-1, TRIM32 becomes upregulated, primarily due to the viral trans-activator of transcription protein (Tat) [247]. This Tat-mediated upregulation of TRIM32 induces proliferation arrest in hNPCs, eventually leading to neurodegeneration [247].

Flaviviruses also encode viral proteins that antagonize TRIM-mediated innate immunity (Figure 5). YFV and DENV NS5 protein have 10 amino acid residues on the N-terminus, which are essential for antagonism of type-I IFN signaling [54,248]. YFV NS5 binds STAT2 only after IFN treatment, and appears to inactivate ISGF3 within the nucleus [54]. Morrison et al. found in DENV NS5 a glycine and a threonine residue within the N-terminus that are required for binding with UBR4 (Ubiquitin Protein Ligase E3 Component N-Recognin 4) to mediate STAT2 degradation [249]. UBR4, a member of the N-recognin family, is a potential E3 ligase that recognizes and degrades proteins containing destabilizing N termini. [250]. UBR4 interacts preferentially with proteolytically-processed DENV NS5, but not with YFV NS5 or WNV NS5 [249]. Although UBR4 does not belong to the TRIM family, it is possible that TRIM members may also be involved in STAT2 degradation by NS5. For example, TRIM23 was identified as an essential factor in YFV replication due to its interaction and poly-Ub of residue K6 on YFV-NS5, promoting binding with STAT2 and inhibition of type-I IFN signaling [54]. Another flavivirus, Japanese encephalitis virus (JEV), induces expression of TRIM21 in human microglial cells, which attenuates JEV-induced antiviral signaling [53]. The study by Manocha et al. demonstrated that TRIM21 overexpression suppressed phosphorylation of IRF3 and activation of IFN-β, while silencing TRIM21 permitted efficient type-I IFN responses in JEV-infected human microglial cells [53]. This study provides evidence that JEV suppress the IFN-I response due to induction of TRIM21.

**Figure 6:**
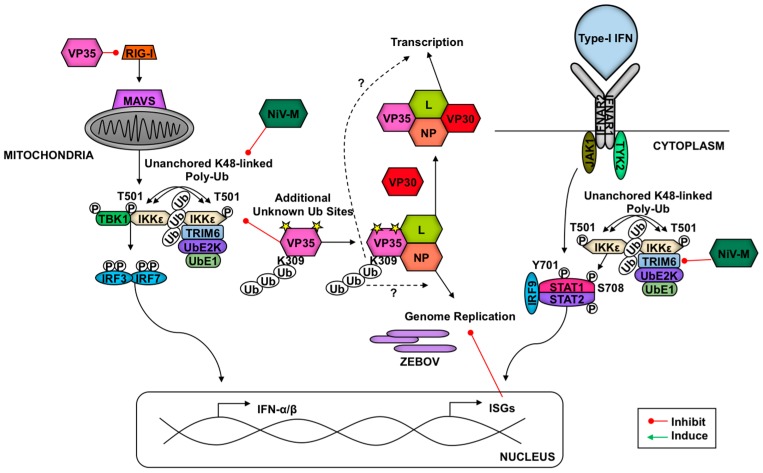
TRIM6 is targeted by Nipah and Ebola viruses to enhance virus replication. Ebola virus (EBOV) inhibits type-I IFN production by multiple mechanisms. EBOV VP35 binds and inhibits RIG-I, IKKε, and TBK-1 to inhibit IFN production. TRIM6 ubiquitinates VP35 on K309 and promotes VP35 activity as the cofactor of the viral polymerase and enhances virus replication. Additional unidentified ubiquitination sites of VP35 exist. Whether TRIM6 enhances EBOV replication by promoting viral genome replication or viral gene transcription is not known. In Nipah virus infection, the viral matrix protein (NiV-M) promotes TRIM6 degradation, resulting in reduced synthesis of K48-linked unanchored polyubiquitin chains, IKKε oligomerization, IKKε-T501 autophosphorylation, IRF3 phosphorylation, and reduced IFN induction. These combined losses confer an impaired host-antiviral response.

Finally, we recently showed that Nipah virus (NiV), a single-stranded negative-sense highly pathogenic RNA virus (*Paramyxoviridae* family, genus *Henipavirus*) that causes fatal diseases in humans [251], can inhibit TRIM6-mediated type-I IFN responses [56] (Figure 6). Mechanistically, the NiV matrix (NiV-M) structural protein, which is required for virus assembly and budding [252,253], targets TRIM6 for degradation. Interestingly, NiV budding requires trafficking of NiV-M from the cytoplasm to the nucleus before reaching the cell membrane for virus assembly [252,253]. The reason for NiV-M trafficking to the nucleus is still unclear, however, a lysine residue (K258) in the NiV-M bipartite nuclear localization signal that is conserved in divergent henipaviruses and is required for trafficking, is critical for the IFN antagonist function [56]. Consistent with this, the matrix proteins of Ghana, Hendra and Cedar viruses were also able to inhibit IFN-β induction [56]. It is currently unknown whether TRIM6 E3-ligase activity affects NiV-M trafficking or whether it directly interferes with NiV-replication. NiV-M-induced TRIM6 degradation did not appear to require the proteasome, however. Although the precise mechanism of TRIM6 degradation remains to be elucidated, inhibitors that recover TRIM6 protein levels could potentially be used as therapeutic drugs against NiV infections. These findings highlight the importance of TRIM6 as an antiviral factor of the type-I IFN system.

### 4.2. Antagonism of TRIMs by DNA Viruses

A variety of DNA viruses also encode viral proteins that interfere with TRIMs. Alternative methods for subverting TRIM-induced innate immune responses can be noted through alterations made to host transcription by hepatitis B virus (HBV). The HBV-encoded protein X (HBx) methylates a single CpG in TRIM22’s promoter region [254]. This viral-mediated epigenetic modification blocks IFN-α/γ-induced IRF1, a transcriptional activator, from binding the TRIM22 promoter and consequently facilitates viral proliferation and escape [255]. In Epstein–Barr virus (EBV), KAP1 (KRAB [Kruppel-Associated Box Domain]-Associated Protein 1)/TRIM28 regulates activation of the viral lytic cycle [256]. In cells undergoing lytic stage activation, KAP1/TRIM28 becomes phosphorylated at serine residue 824 by ataxia telangiectasia mutated (ATM). This post-translational modification impairs KAP1/TRIM28’s restriction factor function and allows EBV to transition from latency to lytic stage [256]. Additionally, the anti-malarial drug chloroquine acts as an activator for ATM by phosphorylating KAP1/TRIM28, which ultimately leads to promotion of the EBV lytic cycle and escape of the virus particles [256].

Mutations in gene expression serve as an additional focal point highlighting the role that TRIMs play in a dysfunctional antiviral response. When a population of 765 HBV-infected individuals was screened, chronic HBV infection was found to correlate to a single T to C silent mutation single nucleotide polymorphism (SNP) in the TRIM22 RING domain [257]. Although the implications to patient outcomes are considerable, additional investigations into the basic biological mechanisms are warranted to discern the importance of these SNPs in TRIM-mediated innate immunity. 

In addition to impairing TRIM gene expression, DNA viruses can mimic host proteins to prohibit TRIM function. The immediate early protein (ICP0) of herpes simplex virus 1 (HSV-1) possesses a RING domain and facilitates the degradation of TRIM27 through ubiquitination [258]. This destruction of TRIM27 is facilitated through the host’s proteasome [258], mimicking the numerous instances of TRIM-mediated restriction. Gammaherpesvirus MHV-68 similarly targets TRIM19 for proteasome-mediated degradation [239]. The IE1 proteins of human cytomegalovirus (HCMV) mimics the coiled-coil domain of TRIMs to recruit and sequester TRIM19 from nuclear bodies thus impairing the activation of IFN responses [239].

## 5. Hijacking of Antiviral TRIMs as a Novel Mechanism to Directly Enhance Virus Replication

So far, most studies on TRIMs have focused on their roles as antiviral factors by directly restricting virus replication or indirectly by inducing antiviral cytokines, as described above. The fact that TRIMs are targeted by viruses for immune evasion further highlights their important roles in protecting the host against infections. However, whether TRIMs may directly act as host factors required for virus replication, or “pro-viral” factors, has not been addressed. Some studies have shown that certain viral antagonists can hijack TRIMs to activate their IFN antagonist activity (e.g., TRIM23 ubiquitinates YFV-NS5 for antagonism of STAT2 function [54]), but these are indirect effects that provide an advantage to the virus by reducing host antiviral responses. Since ubiquitination of viral proteins may positively influence specific steps of the replication cycle, it would not be surprising if TRIMs are involved in directly promoting virus replication by non-degradative ubiquitination of viral proteins.

Indeed, we recently reported the first example of such a role for TRIM6 [259]. As described above in Section 2.6, TRIM6 is involved in antiviral type-I IFN responses by catalyzing the synthesis of unanchored K48-linked polyubiquitin chains that activate IKKε (Figure 2 and Figure 6). Further evidence of TRIM6 as an antiviral factor is highlighted by our findings that the NiV can inhibit IFN-I responses by targeting TRIM6 [56]. Furthermore, knockdown of TRIM6 in lung A549 cell lines and primary human monocyte derived dendritic cells has shown increased replication of multiple viruses, including IAV, EMCV, and Sendai virus (SeV), most probably due to reduced type-I IFN responses [11]. In addition, TRIM6 A549 knockout cells showed reduced IFN responses [259]. Unexpectedly, despite a defect in the IFN response, infectious Ebola virus (EBOV: *Filoviridae* family) replicated less efficiently in TRIM6 knockout cells as compared to parental wild type (WT) cells. This observation raised the question whether TRIM6 may be acting as a pro-viral factor or an enhancer of virus replication. In support of this hypothesis, we found that TRIM6 interacts with EBOV-VP35, which is a major viral IFN antagonist by targeting RIG-I [260,261] and the kinases IKKε and TBK-1 [262]. However, since VP35 also plays a critical role as the cofactor of the virus polymerase [263], TRIM6 could directly affect polymerase function. Mass spectrometry analysis and co-immunoprecipitation assays demonstrated that TRIM6 ubiquitinates VP35 on K309 [259], a lysine residue located on its IFN antagonist domain. Moreover, minigenome reporter experiments showed that TRIM6 can enhance VP35-mediated polymerase activity, and this effect requires the E3-ubiquitin ligase activity of TRIM6. Although VP35 is ubiquitinated in multiple (currently unidentified) residues in addition to K309, the TRIM6-dependent effects on VP35 minigenome activity required an intact K309 residue. VP35 was also able to inhibit RIG-I induced TRIM6-enhanced IFNβ in reporter assays, however the precise mechanism was not elucidated [259]. Collectively, these findings suggest that TRIM6 is a host factor hijacked by EBOV-VP35 for both immune evasion and for promoting virus replication via ubiquitination of VP35 (Figure 6). Future studies will address whether ubiquitination of VP35 regulates virus RNA replication or transcription. It remains to be seen if other TRIMs that are targeted by viruses may also directly enhancing virus replication via ubiquitination of viral proteins.

## 6. Potential Roles of TRIM-mediated Autophagy during Virus Infections

Autophagy (self-eating) is a highly conserved catabolic mechanism through which eukaryotic cells deliver dispensable, or potentially dangerous, cytoplasmic material to lysosomes for degradation [264]. The process is characterized by the formation of autophagosomes, which sequester the cytoplasmic structures targeted for destruction. Autophagy has been linked to a wide range of physiological processes, including cell differentiation and development, the degradation of aberrant structures and turnover of damaged organelles, as well as innate and adaptive immunity [265,266]. A growing number of studies indicate that several TRIMs are linked to autophagy and recent excellent reviews are available on the emerging roles of TRIMs in autophagy [59,60,267,268].

A good example of the potential role of TRIMs in autophagy and virus infections is rhesus TRIM5α, which acts both as a regulator of autophagy by providing a platform for the assembly of activated ULK1 and Beclin 1 (key components of the autophagy regulatory complexes) and as a receptor for selective autophagy [269,270]. In its role as an autophagic cargo receptor, TRIM5α directly recognizes viral capsid sequences via its SPRY domain [158,269,270,271]. This is an example of selective autophagy in mammalian cells, which could occur via direct substrate recognition by TRIMs and connects autophagy with a role in defense against viral pathogens. The rhesus TRIM5 can execute precision autophagy of the HIV-1 capsid. In contrast, the weak affinity of human TRIM5 for the HIV-1 capsid precludes effective precision autophagy. As a consequence, rhesus TRIM5, but not human TRIM5, could contribute to defense against HIV-1 through precision autophagy. Since this recognition depends on the C-terminal region of TRIMs (e.g., SPRY domain of TRIM5α), other types of C-terminal domains on TRIMs could selectively recognize diverse protein targets. Thus, TRIM proteins, as a group, could comprise a class of broad-repertoire, high-fidelity, selective autophagic receptors. Given the breadth of the role of TRIMs in various diseases, it will be important to explore precision autophagy—in addition to bulk autophagy—as a therapeutic target against viral infections.

The proposed role of TRIMs in autophagy raise questions. For example, how many TRIMs act as autophagic receptors and what are their specific targets? Do TRIM proteins function as hubs connecting different signaling pathways or different systems? How is the autophagic role of TRIMs integrated with the other functions of TRIMs, including regulation of gene expression and pro-inflammatory signaling? What is the interplay between the E3 ligase activity of TRIMs and precision autophagy? It is important to determine inhibitory compounds of TRIM proteins for their use as therapeutic tools in infectious diseases. However, because some TRIM proteins have simultaneous dual functions in carcinogenesis and the immune response, it should be considered that putative drugs (inhibitors of some TRIM proteins) for cancer therapy may affect immunological reactions as a side effect. Further detailed analysis of TRIM proteins is needed for their use as novel therapeutics with minimal side effects.

## 7. Conclusions and Future Perspectives

This review highlighted the roles of TRIMs in virus–host interactions. Extensive reports detail TRIM involvement in immune signaling and direct virus restriction. In addition, viral antagonism of TRIMs exemplifies the importance of this protein family in antiviral responses. Despite these advancements, many TRIMs have yet to be characterized. Additionally, the molecular mechanisms underlying TRIM-mediated virus restriction or viral protein-mediated TRIM inhibition are not fully elucidated. The role of TRIMs in regulating poly-Ub chain topology is also of interest. In several examples, including TRIMs 5 [169], 6 [11], 21 [12], and 25 [77], TRIMs facilitate the synthesis of unanchored poly-Ub chains. The relative contribution of TRIMs in synthesizing specifically unanchored poly-Ub chains, versus covalently linked poly-Ub chains, is unclear. Classically, the E2 is considered more important in determining poly-Ub chain characteristics [4,5], but post-translational modifications may influence the decision between covalent and non-covalent linkage [12] as may the choice of the partnering E3. Perhaps TRIMs play a unique role in the synthesis of unanchored chains. In the future, evaluating the factors that regulate E2-TRIM pairing may add an additional layer of complexity to TRIM-mediated regulation and the ubiquitin code. Although one study addressed this question by testing interactions between TRIMs and the E2-conjugases and a few TRIM-E2 pairs were identified [272], the complexity of potential transient interactions, and the possibility of cell-type specific expression for the combination of TRIMs and E2-conjugases makes this task a huge challenge.

Importantly, another question is how poly-Ub chains of unconventional linkages may affect virus replication. For example, unanchored poly-Ub chains can be incorporated into the IAV virion to enable hijacking of the host’s aggresomal pathway to facilitate viral replication [192]. The E2 and E3 enzymes responsible for synthesizing these poly-Ub chains have not been identified, but perhaps IAV hijacks a TRIM to make these poly-Ub chains critical in efficient infection. Therefore, unanchored poly-Ub chains may have both pro-viral and antiviral functions [195] and elucidating how to tip the balance towards antiviral responses may help develop novel antiviral therapies. Additionally, IAV replication via host ubiquitin and aggresome systems relies on the host’s cytoskeletal network [192]. This may further implicate the role of TRIMs in similar pathways as several TRIMs associate with microtubules [18,31]. Many TRIMs assemble into cytoplasmic bodies, which can be dynamic structures as described with TRIM32 [35]. The exact role of cytoplasmic bodies is not well characterized. Possible TRIM-cytoplasmic body functions may include facilitation of signaling complex assembly [11], regulation of active TRIM solubility, and/or generation of autophagosome-like complexes to target proteins for degradation. Viruses can also disrupt or reorganize TRIM-cytoplasmic bodies, further supporting a role in antiviral responses. Understanding how TRIM sub-cellular localization influences activity will also benefit the field.

The applicability of the information garnered from studying TRIM–virus interactions may facilitate the identification of novel antiviral targets. Further studies will advance our understanding of the complexities involved with TRIM signaling such as isoform-specific roles, cell-type specific activity, cytoplasmic body assembly and function, and post-transcriptional modification-induced TRIM function. Finally, future studies will need to address whether other TRIMs, in addition to TRIM6, may be hijacked by viruses to directly enhance replication, acting as pro-viral factors.
